# Evaluation of the Cardiac Effects of Inflammation in Patients with Rheumatoid Arthritis and Spondyloarthritis

**DOI:** 10.5152/eurasianjmed.2025.25777

**Published:** 2025-05-30

**Authors:** Ayşegül Şahin, Atalay Doğru, Mustafa Karabacak

**Affiliations:** 1 Department of Internal Medicine, Süleyman Demirel University Faculty of Medicine, Isparta, Türkiye; 2 Department of Cardiology, Süleyman Demirel University Faculty of Medicine, Isparta, Türkiye

**Keywords:** Echocardiography, myocardial performance index, rheumatoid arthritis, spondyloarthropathy

## Abstract

**Background:**

This study aimed to evaluate cardiac involvement during active inflammation in 2 pathophysiologically different diseases, rheumatoid arthritis (RA) and ankylosing spondylitis (AS), and the change in cardiac involvement with suppression of inflammation after effective treatment.

**Methods:**

The study involved 30 newly diagnosed, active RA and 31 active AS patients in need of biological treatment. The patients were evaluated by the same cardiologist using pulse wave Doppler and tissue Doppler echocardiography at the beginning of the study and after 3 months of treatment. Myocardial performance index (MPI) was obtained by dividing the sum of isovolumetric relaxation time and isovolumetric contraction time, calculated from the recorded Doppler tracing, by ejection time (ET) from the same tracing was calculated separately for both ventricles.

**Results:**

It was found that diastolic dysfunction was present at the time of diagnosis and did not improve after treatment, despite regression in inflammatory values and joint findings. In the pre-treatment period, MPI was found to be higher than the normal value range in both groups. The MPI was 0.5 ± 0.07 for RA patients and 0.51 ± 0.1 for AS patients. No significant difference was found between the 2 groups before treatment (*P* = .697). In the post-treatment evaluation, no significant difference was found in RA and AS patients compared to pretreatment.

**Conclusion:**

Inflammation-induced cardiac involvement may develop in both RA and AS patients, despite their different pathophysiologic pathways. Longer follow-up periods are necessary for the improvement of inflammation-induced diastolic dysfunction and MPI values compared to joint findings used in activation parameters.

## Introduction

**Main Points**

- Diastolic dysfunction is indicative of a significant inflammatory process that occurs in the initial stages.
- Compared to joint symptoms and acute phase response of inflammation, the cardiac effects may recover at a later stage.
- The use of MPI allows for the evaluation of the impact of inflammation on cardiac function in rheumatologic diseases.

Rheumatoid arthritis (RA) and ankylosing spondylitis (AS) are both inflammatory diseases, but they have different pathophysiological mechanisms.^1^ In addition to joint involvement, rheumatologic diseases may affect other organ systems, including the cardiovascular system. Research has shown that both rheumatologic diseases and high levels of inflammation can lead to vascular and cardiac pathologies. Cardiac involvement in RA can manifest in various ways, including pericarditis, coronary arteritis, myocarditis, valvular heart disease, conduction abnormalities, and pulmonary hypertension. However, the mostly asymptomatic nature of cardiac involvement in RA, the lack of distinctive auscultatory findings and typical symptoms, and non-specific changes on electrocardiography (ECG) can lead to delays in diagnosis.^2^^,^^3^ Conduction abnormalities are the most common cardiac symptom observed in AS patients, occurring more frequently than other cardiac findings such as valvular insufficiency. Echocardiographic findings in patients with AS have shown abnormalities in myocardial function. Diastolic dysfunction resulting from decreased left ventricular (LV) compliance is the most common and significant type.^4^^,^^5^

A plethora of studies have identified numerous pathways implicated in the pathophysiology of cardiac involvement in rheumatological diseases. Specifically, calcium handling abnormalities, indices of P-wave dispersion increase, endothelial dysfunction, autonomic nervous system (ANS) dysfunction with increased sympathetic outflow, and renin angiotensin system activation have been observed in conjunction with elevated tumor necrosis factor- alpha (TNF-α) and IL-6 cytokines.^6^ Inflammation is known to target sarcomeric proteins, fibrinogen and vimentin, resulting in the formation of atherosclerotic plaques. Consequently, endothelial dysfunction and myocardial tissue damage ensue. Consequently, the initial manifestation is that of diastolic dysfunction, progressing to heart failure with a preserved ejection fraction.^7^, ^8^

Echocardiography is an easily accessible, non-invasive, rapid, and safe method that provides comprehensive information on cardiac anatomy (volumes, geometry, and mass), wall motion, and valve function.^9^ Myocardial performance index (MPI) is a simple alternative method obtained by Doppler echocardiographic methods, which can be calculated separately for both ventricles, quantitatively shows global (systolic and diastolic) ventricular functions, and is not affected by ventricular geometry. Tei Chuwa first described MPI in 1995 as the sum of isovolumetric relaxation time (IVRT) and isovolumetric contraction time (IVCT) divided by ejection time (ET). Numerous studies, including those on heart failure, acute myocardial infarction, hypertension, and diabetes mellitus, have found that increased MPI is associated with morbidity and mortality.^10^ In a study conducted in patients with Behçet’s disease, which is a systemic vasculitis involving arteries and veins, it was demonstrated that MPI in the right ventricle was higher than in healthy controls. Therefore, non-invasive MPI was considered to have prognostic value in managing the disease during the asymptomatic vasculitis period of Behçet’s disease.^11^ A study conducted with patients who have scleroderma found that LV MPI was higher compared to the control group.^12^

The aim of this study was to evaluate cardiac involvement using echocardiography during active inflammation in 2 pathophysiologically different diseases, RA and AS, and the change in cardiac involvement with suppression of inflammation after effective treatment.

## Materials and Methods

### Patients and Method

The study involved 30 patients diagnosed with RA based on the 2010 ACR/EULAR classification criteria^13^ and 31 patients diagnosed with AS based on the 2009 ASAS classification criteria.^14^ Upon admission, sociodemographic characteristics, comorbidities, laboratory parameters, pulse rate per minute, and systolic and diastolic blood pressures were recorded. Disease activity was assessed using composite scales before treatment initiation and at the 3-month follow-up. Patients aged 18-65 years with moderate or high disease activity according to the composite disease scales, without a history of cardiac disease or drug use, without hypertension or diabetes mellitus, newly diagnosed RA, and active AS patients in need of biological treatment were included in the study, and appropriate treatments were started. At the 3-month follow-up, patients who were evaluated as non-responders according to composite disease scales were excluded from the study.

The study comprised 30 patients diagnosed with RA who had not received biological treatment. The patients in this group received only low-dose steroids and DMARD therapy, predominantly methotrexate, with leflunomide administered in cases of adverse effects or contraindications, and as a monotherapy. After three months of treatment, echocardiographic measurements were performed again. In the AS group, 31 patients who did not respond to NSAID treatment and required biological treatment were included in the study. Patients who were undergoing anti-TNF therapy as a biological treatment were included in the study. Patients receiving biosimilar drug therapy were also included in the study. Due to the limited number of patients receiving Anti IL-17 and JAK inhibitor therapy, these patients were excluded from the study. Echocardiography was performed prior to the commencement of biological therapy and after three months of therapy. The following biological agents were administered: adalimumab (12 patients), certolizumab (8 patients), etanercept (7 patients), infliximab (3 patients) and golimumab (1 patient).

The disease activity of RA patients was evaluated using the Disease Activity Score (DAS)-28 erythrocyte sedimentation rate (ESR) and C-reactive protein (CRP). The DAS28 comprises the number of 28 swollen joints, the number of 28 tender joints, ESR or CRP levels, and an assessment of disease activity or general health using a visual analog scale. The disease activity level is evaluated as remission (DAS28 < 2.6), low (2.6 ≤ DAS28 < 3.2), moderate (3.2 ≤ DAS28 ≤ 5.1), or high (DAS28 > 5.1). A good response is defined as an improvement of more than 1.2 units in the DAS28 score, resulting in a subsequent DAS28 score of 3.2 or less. A moderate response is defined as an improvement of more than 0.6 and up to 1.2 in the DAS28 score, with a resulting DAS28 score of more than 3.2 and up to 5.1. An unresponsive result is defined as a DAS28 score improvement of 0.6 or less, with a resulting DAS28 score of more than 5.1.^15^

The Bath Ankylosing Spondylitis Disease Activity Index (BASDAI) and Ankylosing Spondylitis Disease Activity Score (ASDAS) were used to evaluate the disease activities of AS patients. The BASDAI is a disease activity assessment scale consisting of 6 items filled out by the patient. The first 5 questions use a numerical response scale (0-10) or VAS (0-10 cm) ranging from “none” to “very severe”. Question 6 determines the duration of morning stiffness on a time scale of 0-2 hours. An active disease is defined as a score of 4 or more. The ASDAS is an AS assessment scale that has become more frequently preferred in recent years and contains inflammatory markers such as ESR or CRP. According to ASDAS-CRP, disease activity is defined as inactive if <1.3, low if <2.1, high if <3.5, and very high if >3.5. A clinically important improvement is reported as an increase of at least 1.1 units in the ASDAS score, while a major improvement is reported as an increase of at least 2.0 units.^16^^,^^17^

The patients in the study were evaluated by the same cardiologist using echocardiography at the beginning of the study and after 3 months of treatment.

Blood samples were collected from the patients in the morning after an 8-hour fast. Erythrocyte sedimentation rate was measured using the Vision C device spectrophotometrically, and CRP was measured using the Roche device nephelometrically.

Artificial intelligence assisted technologies (such as Large Language Models, chatbots, or image generators) were not used in the production of the manuscript. All participants provided informed consent, and the the Ethics Committee for Clinical Research of Süleyman Demirel University Faculty of Medicine (Approval number 2021-23/356; Date: 23.12.2021) approved the study protocol.

### Echocardiography

Transthoracic echocardiography was performed in the Department of Cardiology’s echocardiography laboratory using a high-resolution Philips IE 33 imaging system (Andover, Massachusetts, USA) with transducers arranged at different frequencies (2.5-3.5 MHz). Measurements were taken at the end of expiration during normal breathing while the patient was at rest and in the lateral decubitus position. In accordance with recent recommendations from guidelines,^9^ the same skilled specialist took the measurements while being blind to patient information. Two-dimensional and M-mode echocardiographic testing were performed on all patients using standardized recording methodology and measurements. The data were analyzed by averaging 3 consecutive measurements taken with ECG recording.

The LV end-systolic diameter and end-diastolic diameter, septum and posterior wall thickness, and left atrial diameter were measured using pulse wave Doppler echocardiography from the parasternal long axis. Additionally, a 2-dimensional echocardiographic examination was conducted to evaluate wall motion, valve structure and function, and pericardial pathologies. The images of the LV during systole and diastole were determined to be the smallest and widest, respectively. The LV ejection fraction (EF) was calculated using the Modified Simpson’s Method on apical 2- and 4-chamber images. The LV filling pattern was examined using a pulse wave Doppler by placing a sample volume 1 cm above the mitral valve cusps in the apical 4-chamber image. The peak velocities of early (E) and late diastolic flows (A) and the mitral E/A ratio were calculated from the recordings. The deceleration time (DT) of the early diastolic mitral flow was also measured. The sample volume was positioned between the outflow tract of the LV and the mitral valve in the apical 5-cavity view. Myocardial performance index was obtained by dividing the sum of IVCT and IVRT, calculated from the recorded Doppler tracing, by ET from the same tracing. The MPI enables the evaluation of both RV and LV and provides information on both systolic and diastolic functions. The mean MPI values were 0.39 ± 0.05 for LV and 0.28 ± 0.04 for RV. The velocities of Ea (Mitral Annular E wave) and Aa (Mitral Annular A wave) were calculated by averaging the medial and lateral regions of the mitral annulus from the apical 4-cavity image with myocardial tissue Doppler echocardiography. The Ea/Aa ratio and E/Ea were also calculated.

### Statistical Analysis

The statistical analysis was performed using SPSS 20.0 software (IBM SPSS Corp.; Armonk, NY, USA). Descriptive statistics were used to present categorical data as frequency (percentage ratio) and proportional scale data as mean ± SD. The normal distribution of continuous numerical data was tested using the Kolmogorov–Smirnov method, and parametric methods were used for comparisons between groups and repeated measurements. The Student’s *t*-test was used to compare 2 independent groups, and the paired *t*-test was used to compare 2 repeated measures. Chi-square analysis was used to determine the relationships between categorical data. It was considered significant when the *P*-value was less than .05, with a Type-I error rate of 5% throughout the study.

## Results

The study analyzed 30 patients with RA and 31 patients with AS. Twenty-three (76%) of RA patients and 23 (74%) of AS patients were female. When evaluated according to body mass index, both patient groups were similar. While newly diagnosed RA patients were included in the study, the median disease age of AS patients was calculated at 36 (3-96) months. The DAS-28 CRP value of RA patients was 4.7 ± 1.0 before treatment and 3.0 ± 1.1 after treatment, and statistical significance was observed. In AS patients, ASDAS CRP was 3.2 ± 0.7 prior to treatment and 1.9 ± 0.9 following treatment; BASDAI was 5.5 ± 1.8 prior to treatment and 2.7 ± 1.8 following treatment. Systolic and diastolic blood pressure and pulse rate were similar in both patient groups before and after treatment (Table 1).

No significant changes were found in the heart valves of RA patients before and after treatment. Pre-treatment EF was 63.33 ± 2.39, and no significant difference was obtained when compared with post-treatment. Pulse-wave Doppler echocardiography showed no significant changes in LVESC, LVEDC, mitral E and A waves, the E/A ratio, or DT. Tricuspid annular plane systolic excursion (TAPSE) was also assessed and found to be 23.98 ± 6.98 before treatment and 24.99 ± 5.94 after treatment, with no significant difference observed between pre- and post-treatment. In RA patients, IVRT, IVCT, and ET were assessed via echocardiography, and the MPI was calculated. The MPI in RA patients was 0.5 ± 0.07 before treatment and 0.51 ± 0.08 after treatment. No significant difference in MPI was observed before and after treatment in RA patients (Table 2).

When patients were split into seropositive and seronegative groups, there was no significant alteration seen in echocardiographic data before and after treatment. Upon evaluating the correlation between disease activation parameters and echocardiographic parameters before and after treatment, a significant negative correlation was found between ESR and IVRT and ET (*P* values of .04-.03, respectively) and between pulmonary artery pressure (PAP) and CRP and DAS-28 CRP (*P* values of .04-.04, respectively).

Pulse wave and tissue Doppler echocardiography values of AS patients showed no significant change before and after treatment. The pre-treatment MPI of patients evaluated in IVRT, IVCT, and ET was 0.51 ± 0.1. In the post-treatment echocardiography, MPI was 0.52 ± 0.07. Myocardial performance index measurement by tissue Doppler echocardiography was similar before and after treatment. In the correlation of inflammation parameters with echocardiography data, significant negative correlations were observed between TAPSE and ESR and CRP (*P* values of .003-.02, respectively) and between right ventricular thickness and ESR, CRP, and ASDAS CRP (*P* values of .001-.02-.02, respectively)(Table 3).

Pre-treatment MPI values of RA and AS patients were 0.5 ± 0.07 and 0.51 ± 0.1, respectively, with no statistically significant difference (*P*-value = .69). There was no significant difference in post-treatment MPI values for both diseases.

## Discussion

Inflammation can cause cardiovascular damage through various mechanisms.^18^ Rheumatologic diseases can lead to cardiac involvement due to increased inflammation. The study evaluated the cardiac effects of inflammation in patients with RA and AS, 2 diseases with different pathophysiologic mechanisms. Elevated MPI values were found in both patient groups compared to the healthy population (MPI normal value for LV was 0.39 ± 0.05). According to the literature, elevated MPI values were observed to be associated with inflammation. Active inflammation causes elevated MPI in patients, regardless of the disease group.

In patients with RA, differences in both cardiac morphology and cardiac function were found in comparison to normal subjects, even in the absence of underlying heart disease. Aslam et al^19^ conducted a meta-analysis on patients diagnosed with RA and found that the RA patient group had an elevated A wave, a decreased E/A ratio, and prolonged IVRT compared to the control group. This led to the conclusion that RA is associated with diastolic dysfunction. In another study, which followed RA patients for 5 years, a decrease in the E/A ratio was observed along with a more increased A wave compared to the general population.^20^ According to the study, the normal range for the E wave was 86 ± 16 cm/s, the A wave was 56 ± 13 cm/s, and the E/A ratio was reported as 1.6 ± 0.5.^21^ Studies using tissue Doppler echocardiography, a relatively new technique for evaluating global or regional systolic and diastolic ventricular function, found that RA patients had a low Ea wave, a high Aa wave, and a low Ea/Aa ratio compared to the control group. This suggests diastolic dysfunction.^22^^,^^23^ The study found an elevation in the A wave and a decrease in the E/A ratio before treatment, consistent with the literature and supporting diastolic dysfunction. No significant changes in the E and A waves or the E/A ratio were observed in the post-treatment evaluation. In this study of newly diagnosed RA patients, it was found that diastolic dysfunction was present at the time of diagnosis and did not improve after treatment, despite regression in inflammatory values and joint findings.

A review evaluating LV diastolic functions by echocardiography in AS patients found a low E/A ratio, prolongation in DT, and prolongation in IVRT. It was suggested that there is a disorder in LV diastolic function similar to that in RA patients.^24^ In the pre-treatment evaluation, a prolongation in the A wave, a decrease in the E/A ratio compared to normal, and a shortening in the DT compared to the normal range were observed.

The MPI is a crucial parameter for evaluating both systolic and diastolic function. An increase in MPI indicates irregularities in these functions.^25^ Studies have shown that MPI increases in heart failure, coronary artery disease, and myocardial infarction. It has been reported to be useful in rheumatologic diseases and in the evaluation of the cardiac effects of chemotherapeutic drugs.^26^ The normal range of MPI for LV was reported as 0.39 ± 0.05 in studies.^27^ .In a study comprising 32 patients with RA and 32 healthy controls, LV MPI was calculated as 0.44 ± 0.11 in patients with RA, which was significantly higher than in the control group.^28^ Levendoğlu et al^29^ similarly found the MPI value to be higher in RA patients than in the control group. When MPI was evaluated in AS patients, Okan et al^30^ found that MPI was 0.47 ± 0.07 in AS patients, which was significantly higher than in the control group. In a study conducted by Moyssakis et al,^31^ it was found that MPI was significantly higher in AS patients. This study found that LV MPI was 0.5 ± 0.07 in RA patients and 0.51 ± 0.1 in AS patients before treatment. The MPI was found to be high in both disease groups compared to the normal value range (MPI normal value was 0.39±0.05) and remained similarly high in the third month after treatment. Although moderate to good treatment success was achieved in both diseases in composite indices evaluating disease activation, this was not reflected in cardiac terms. This suggests that the duration of treatment was not sufficient to provide cardiac benefit. It is thought that longer treatment and follow-up periods are needed compared to joint and acute phase responses in order to see regression in cardiac involvement in patients.

The study has some limitations. One limitation of the study is the absence of a healthy control group. Although detailed echocardiographic data for the healthy control group is available in the literature, its inclusion was not possible. Additionally, the study is limited by the small number of patients and the short follow-up period. For more accurate data on heterogeneous rheumatologic diseases, studies with larger patient groups and longer follow-up periods are necessary. The study’s strengths lie in the evaluation of 2 diseases with different pathophysiologic mechanisms by the same cardiologist, both during a period of high inflammation and after three months of treatment.

In conclusion, the study found that MPI was elevated in newly diagnosed RA patients and active AS patients who required biologic therapy, compared to the normal value range, at the first evaluation before treatment initiation. This suggests that inflammation-induced cardiac involvement may develop in both diseases, despite their different pathophysiologic pathways. In both RA and AS patients, diastolic dysfunction was found in the pre-treatment evaluation. The presence of both MPI elevation and diastolic dysfunction in the initial echocardiograms suggests a correlation with the severe inflammation periods experienced by patients in both groups. Myocardial performance index is a crucial parameter for assessing RV and LV function and can be used to evaluate the impact of inflammation on cardiac function in rheumatologic diseases. However, it is important to note that longer follow-up periods are necessary for the improvement of inflammation-induced diastolic dysfunction and MPI values compared to joint findings used in activation parameters.

## Figures and Tables

**Table 1.** Sociodemographic Characteristics and Disease Activity Status of Patients

Parameters	RA (n: 30)			AS (n: 31)		*P*
Age, years	46.9 ± 11.3			42.8 ± 11.4		NS
Woman, n (%)	23 (76%)			23 (74%)		NS
BMI, kg/m^2^	25.8 ± 4.4			27.7 ± 5.5		NS
Disease duration, month	New diagnosis			36 (3-96)		
RF-ACPA positive, n (%)	16 (53%)					
HLA B27 positive, n (%)				21 (67%)		
	**Before Tx**	**After Tx**	***P***	**Before Tx**	**After Tx**	***P***
DAS28 CRP	4.7 ± 1.0	3.0 ± 1.1	<.001*			
ASDAS CRP				3.2 ± 0.7	1.9 ± 0.9	<.001*
BASDAI				5.5 ± 1.8	2.7 ± 1.8	<.001*
SBP, mmHg	129.0 ± 7.8	128.1 ± 6.7	NS	134.5 ± 9.4	131.8 ± 7.6	NS
DBP, mmHg	79.9 ± 4.6	79.3 ± 3.5	NS	82.5 ± 4.3	79.8 ± 4.6	NS
Pulse, /min	85.6 ± 12	81.13 ± 17	NS	81.5 ± 14	82.7 ± 8.6	NS

ACPA, Anti-citrullinated protein antibodies; AS, ankylosing spondylitis; ASDAS, ankylosing spondylitis disease activity score; BASDAI, bath ankylosing spondylitis disease activity index; BMI, body mass index; CRP, C-reactive protein; DAS28, disease activity score; DBP, diastolic blood pressure; NS, not significant; RA, rheumatoid arthritis; SBP, systolic blood pressure.

*Significant at 0.05 level according to paired Student’s *t*-test.

**Table 2.** Pulse Wave Doppler and Tissue Doppler Echocardiography Findings in RA Patients Before and After Treatment

RA	Before Treatment	After Treatment	*P*
Parameters	Mean ± SD (n = 30)	Mean ± SD (n = 30)	
Pulse Wave Doppler Echocardiography			
LVESD, mm	42.56 ± 8.76	43.43 ± 4.08	.567
LVEDD, mm	25.81 ± 5.89	27.17 ± 3.46	.235
EF, %	63.33 ± 2.39	63.50 ± 2.33	.573
E, cm/sec	81.53 ± 18.93	76.57 ± 19.17	.231
A, cm/sec	81.77 ± 23.79	82.48 ± 17.36	.787
E/A	1.04 ± 0.28	0.96 ± 0.3	.155
DT, msec	132.3 ± 49.39	122.2 ± 45.28	.437
PAP, mmHg	21.6 ± 13.62	22.04 ± 16.13	.838
TAPSE, mm	23.98 ± 6.98	24.99 ± 5.94	.552
ASD, mm	28.99 ± 3.06	30.27 ± 3.39	.059
ADD, mm	26.07 ± 2.97	27.57 ± 2.86	.010*
IVRT, ms	85.13 ± 10.93	88.27 ± 9.96	.224
IVCT, ms	49.7 ± 9.27	46.63 ± 10.31	.219
ET, ms	272.87 ± 31.31	266 ± 27.69	.271
MPI	0.5 ± 0.07	0.51 ± 0.08	.595
Tissue Doppler Echocardiography			
Ea, cm/sec	9.54 ± 2.41	8.63 ± 2.24	.121
Aa, cm/sec	8.84 ± 1.93	10.87 ± 13.14	.403
Ea/Aa	1.13 ± 0.39	1.09 ± 0.47	.600
Sa, cm/sec	8.12 ± 5.94	6.45 ± 1.41	.133
IVRT, ms	85.73 ± 12.08	86.83 ± 11.42	.678
IVCT, ms	49.77 ± 13.67	53.73 ± 12.91	.254
ET, ms	273.53 ± 55.53	281.87 ± 26.57	.418
MPI	0.54 ± 0.27	0.50 ± 0.08	.496

A, late diastolic flow peak velocity; Aa, late diastolic filling mitral annular wave; ADD, aortic diastolic diameter; ASD, aortic systolic diameter; E, early diastolic flow peak velocity; Ea, early diastolic filling mitral annular wave; EF, ejection fraction; ET, ejection time; IVCT, isovolumetric contraction time; IVRT, isovolumetric relaxation time; MPI, myocardial performance index; LVEDD, left ventricle end-diastolic diameter; LVESD, left ventricle end-systolic diameter; PAP, pulmonary artery pressure; RA, rheumatoid arthritis; Sa, systolic mitral annular wave; TAPSE, tricuspid annular plane systolic excursion.

*Significant at 0.05 level according to paired Student’s *t*-test.

**Table 3.** Pulse Wave Doppler and Tissue Doppler Echocardiography Findings in AS Patients Before and After Treatment

AS	Before Treatment	After Treatment	*P*
Parameters	Mean ± SD (n = 31)	Mean ± SD (n = 31)	
Pulse Wave Doppler Echocardiography			
LVESD, mm	44.56 ± 4.31	42.27 ± 9.86	.241
LVEDD, mm	25.89 ± 6.41	26.63 ± 6.6	.673
EF, %	62.09 ± 3.10	62.14 ± 2.84	.161
E, cm/sec	85.74 ± 17.51	81.03 ± 22.88	.354
A, cm/sec	81.22 ± 18.58	79.94 ± 23.27	.718
E/A	1.07 ± 0.26	1.07 ± 0.36	.956
DT, msec	142.65 ± 37.95	131.35 ± 58.85	.373
PAP, mmHg	23.75 ± 12.67	23.89 ± 13.27	.943
TAPSE, mm	25.26 ± 5.53	24.58 ± 7.22	.689
ASD, mm	29.5 ± 3.43	29.59 ± 3.9	.879
ADD, mm	26.29 ± 2.64	25.94 ± 6.05	.756
IVRT, ms	86.52 ± 11.4	86.03 ± 11.39	.874
IVCT, ms	50.58 ± 12.12	49.1 ± 9.64	.573
ET, ms	274.23 ± 35.33	263.81 ± 25.08	.119
MPI	0.51 ± 0.1	0.52 ± 0.07	.765
Tissue Doppler Echocardiography			
Ea, cm/sec	9.2 ± 3.2	9.25 ± 2.51	.945
Aa, cm/sec	8.12 ± 3.01	7.51 ± 2.53	.401
Ea/Aa	1.2 ± 0.38	1.38 ± 0.59	.113
Sa, cm/sec	6.15 ± 0.93	6.19 ± 1.4	.897
IVRT, ms	83.81 ± 23.75	85.81 ± 9.47	.685
IVCT, ms	53.9 ± 10.3	53 ± 11.6	.725
ET, ms	285.16 ± 26.74	272.65 ± 55.18	.220
MPI	0.49 ± 0.11	0.5 ± 0.09	.553

A, late diastolic flow peak velocity; Aa, late diastolic filling mitral annular wave; ADD, aortic diastolic diameter; AS, ankylosing spondylitis; ASD, aortic systolic; E, early diastolic flow peak velocity; Ea, early diastolic filling mitral annular wave; EF, ejection fraction; ET, ejection time; IVCT, isovolumetric contraction time; IVRT, isovolumetric relaxation time; LVEDD, left ventricle end-diastolic diameter; LVESD, left ventricle end-systolic diameter; MPI, myocardial performance index; PAP, pulmonary artery pressure; Sa, systolic mitral annular wave; TAPSE, tricuspid annular plane systolic excursion.

## Data Availability

The data that support the findings of this study are available on request from the corresponding author.
